# A combined planning approach for improved functional and esthetic outcome of bimaxillary rotation advancement for treatment of obstructive sleep apnea using 3D biomechanical modeling

**DOI:** 10.1371/journal.pone.0199956

**Published:** 2018-08-09

**Authors:** Robert Frey, Barbora Gabrielova, Evgeny Gladilin

**Affiliations:** 1 Department of Oral and Maxillofacial Surgery, Seegartenklinik, Adenauerplatz 4/1, 69115 Heidelberg, Germany; 2 BioQuant, University Heidelberg, Im Neuenheimer Feld 267, 69120 Heidelberg, Germany; 3 Division of Theoretical Bioinformatics, German Cancer Research Center, Berliner Str. 41, 69120 Heidelberg, Germany; Ohio State University, UNITED STATES

## Abstract

In recent years, bimaxillary rotation advancement (BRA) has become the method of choice for surgical treatment of obstructive sleep apnea (OSA). As dislocation of the jaw bones affects both, airways and facial contours, surgeons are facing the challenge of finding an optimal jaw position that allows for the reestablishment of normal airway ventilation and esthetic surgical outcome. Owing to the complexity of the facial anatomy and its mechanical behavior, individual planning of surgical OSA treatment under consideration of functional and esthetic aspects presents a challenge that surgeons typically approach in a non-quantitative manner using subjective evaluation and clinical experience. This paper describes a framework for individual planning of OSA treatment using bimaxillary rotation advancement, which relies on computational modeling of hard and soft tissue mechanics. The described framework for simulation of functional and esthetic post-surgery outcome was used in 10 OSA patients. Comparison of the simulation results with post-surgery data reveals that biomechanical simulation provides a reliable estimate for post-surgery facial tissue behavior and antero-posterior airway extension, but fails to accurately describe a surprisingly large lateral stretch of the velopharyngeal region. This discrepancy is traced back to anisotropic effects of pharyngeal muscles. Possible approaches to improving the accuracy of model predictions and defining sharp criteria for optimizing combined OSA planning are discussed.

## Introduction

Reduced lung ventilation and resulting blood oxygenation due to obstructive sleep apnea (OSA) is known to be related to a plethora of pathological syndromes including musculoskeletal, heart and mental disorders [1–8]. Surgical treatment of OSA using bimaxillary rotation advancement (BRA) with counter clockwise rotation aims to mechanically widen constricted airways which provides remedy for OSA symptoms. The efficacy of BRA for surgical treatment of OSA has been demonstrated in a number of previous works [9–11]. Surgical success of OSA treatment is often evaluated on the basis the Sher’s criterion [12], i.e., a greater than 50% reduction of the apnea–hypopnea index (AHI) and/or an AHI of less than 20 events per hour, and its modifications [13]. *Zinser et al*. report an average reduction of AHI from 47.9 ± 15.6 before to 5.6 ± 2.1 after BRA [14]. However, tangible quantitative criteria for OSA diagnostics and individual surgery planning under consideration of both functional and esthetic aspects are not yet well established. Previous experimental and computational works have indicated a causal relationship between geometrical and mechanical properties of airway walls and stability of pharyngeal airflow [15–18]. Narrow and mechanically compliant airway walls cause turbulences in pharyngeal airflow that, in turn, exert negative pressure on soft tissue walls resulting in their further collapse [19]. Bimaxillary advancement is capable to efficiently widen constricted airways, especially in the velopharyngeal region, which reduces the risk of irregular jet-like airflow [20, 21]. In the absence of reliable tools for individual planning of bimaxillary advancement, surgeons tend to undertake maximal admissible bone displacement to achieve a therapeutically sufficient extension of the constricted pharyngeal regions. Advancing the maxillo-mandibular complex by 1cm or greater is frequently suggested in the literature as a common rule for surgical treatment of OSA [22, 23]. However, large maxillo-mandibular displacements may have a strong impact on patients’ facial contours, occasionally causing a pronounced mid-face elongation. While physical mechanisms of OSA have been previously investigated in a number of isolated experimental and computer modeling studied, little has been done, to date, to integrate these findings into routine planning and customization of surgical OSA treatment. In our previous works [24], a general approach to anatomy- and physics-based modeling of cranio-maxillofacial surgery interventions was presented. Here, we extend this approach to quantitatively assess the impact of jaw dislocation on facial and pharyngeal soft tissues. This work presents a methodological framework for customized planning of bimaxiallary rotation advancement resulting in the first reported feasibility study involving post-surgical evaluation of functional and esthetic outcome.

## Methods

### Participant information and study design

This study deals with comparative analysis of pre/post facial and pharyngeal soft tissue in 10 patients who underwent the BRA treatment performed by the first author (R.F.). Participant information and pre-/post-surgery measurements of AHI, velo- (VPX) and laryngopharyngeal (LPX) cross section areas and dimensions are summarized in Table 1. Imaging of patient’s head and assessment of OSA symptoms were performed 2-4 weeks before and repeated 12-24 weeks after surgery.

**Table 1:** Participant demographic and biometric information, maxillo-mandibular impactions (Mxi) / protrusions (Mxp, Mdp) measurements of pre-/post-surgery AHI, the narrowest velo- and laryngopharyngeal cross section of pre-/post-surgical airways including AP/Lat diameters and cross section areas.

									Min. VPX cross section						Min. LPX cross section					
				MMA [cm]			AHI/h		A [cm^2^]		AP [cm]		Lat [cm]		A [cm^2^]		AP [cm]		Lat [cm]	
N	Age	Sex	BMI	Mxi	Mxp	Mdp	pre	post	pre	post	pre	post	pre	post	pre	post	pre	post	pre	post
1	37	M	27.6	0.3	0.3	1.2	34.3	4.7	0.9	1.9	0.6	1.0	1.9	2.1	3.4	4.9	1.8	2.5	2.5	3.2
2	25	F	25.7	0.3	0.3	1.0	20.0	0.5	1.7	2.9	0.7	1.1	2.5	3.1	1.6	4.3	1.2	1.9	2.6	3.1
3	53	M	38.6	0.3	0.6	1.3	59.1	2.8	0.4	1.3	0.3	0.8	1.4	2.4	3.1	4.5	1.3	1.7	3.2	3.7
4	37	M	28.9	0.3	0.5	1.0	43.1	14.6	0.4	1.6	0.4	0.9	1.2	1.9	1.9	4.2	1.3	2.3	2.1	3.2
4	37	M	28.9	0.3	0.5	1.0	43.1	14.6	0.4	1.6	0.4	0.9	1.2	1.9	1.9	4.2	1.3	2.3	2.1	3.2
5	25	F	24.5	0.3	0.7	1.0	12.5	7.0	3.1	5.2	1.1	1.8	2.8	3.5	2.7	3.1	1.7	1.8	2.5	2.8
6	30	F	25.1	0.3	0.6	1.1	30.8	4.8	0.8	1.5	0.5	0.9	1.5	2.0	2.0	2.9	1.4	1.6	2.3	2.7
7	49	M	33.2	0.3	0.3	1.2	27.8	10.8	1.2	2.9	0.7	1.2	2.0	2.8	1.9	4.1	1.3	2.4	2.1	3.2
8	55	F	32.5	0.3	0.5	0.9	65.2	5.1	0.5	1.3	0.5	0.9	1.1	1.7	1.6	1.9	1.0	1.4	1.6	1.7
9	29	F	24.7	0.3	0.6	0.9	88.2	36.6	2.1	3.6	0.8	1.4	2.4	3.3	4.0	5.5	1.3	1.8	3.6	3.9
10	55	F	29.4	0.3	0.3	1.1	76.0	26.5	0.5	1.7	0.3	0.8	1.6	2.4	0.6	0.9	1.0	0.9	1.0	1.3
mean	39.5	-	29.0	0.3	0.5	1.1	45.7	11.3	1.2	2.4	0.6	1.1	1.8	2.5	2.3	3.9	1.3	1.9	2.4	3.1
std	12.4	-	4.6	0.0	0.2	0.1	25.2	11.6	0.9	1.3	0.2	0.3	0.6	0.6	1.0	1.4	0.3	0.5	0.7	0.8
min	25.0	-	24.5	0.3	0.3	0.9	12.5	0.5	0.4	1.3	0.3	0.8	1.1	1.7	0.6	0.9	1.0	0.9	1.0	1.3
max	55.0	-	38.6	0.3	0.7	1.3	88.2	36.6	3.1	5.2	1.1	1.8	2.8	3.5	4.0	5.5	1.8	2.5	3.6	3.9

### Ethics statement

This study was approved by the Seegarten Clinic Ethics Committee, approval no. PFS21002-34. All procedures were carried out in accordance with the ethics standards of the responsible committee on human experimentation and with the Helsinki Declaration revised in 2008. Participating patients were informed about assessment and usage of their anonymized data for research purposes in verbal and written form.

### Surgical techniques

**Fig 1:**
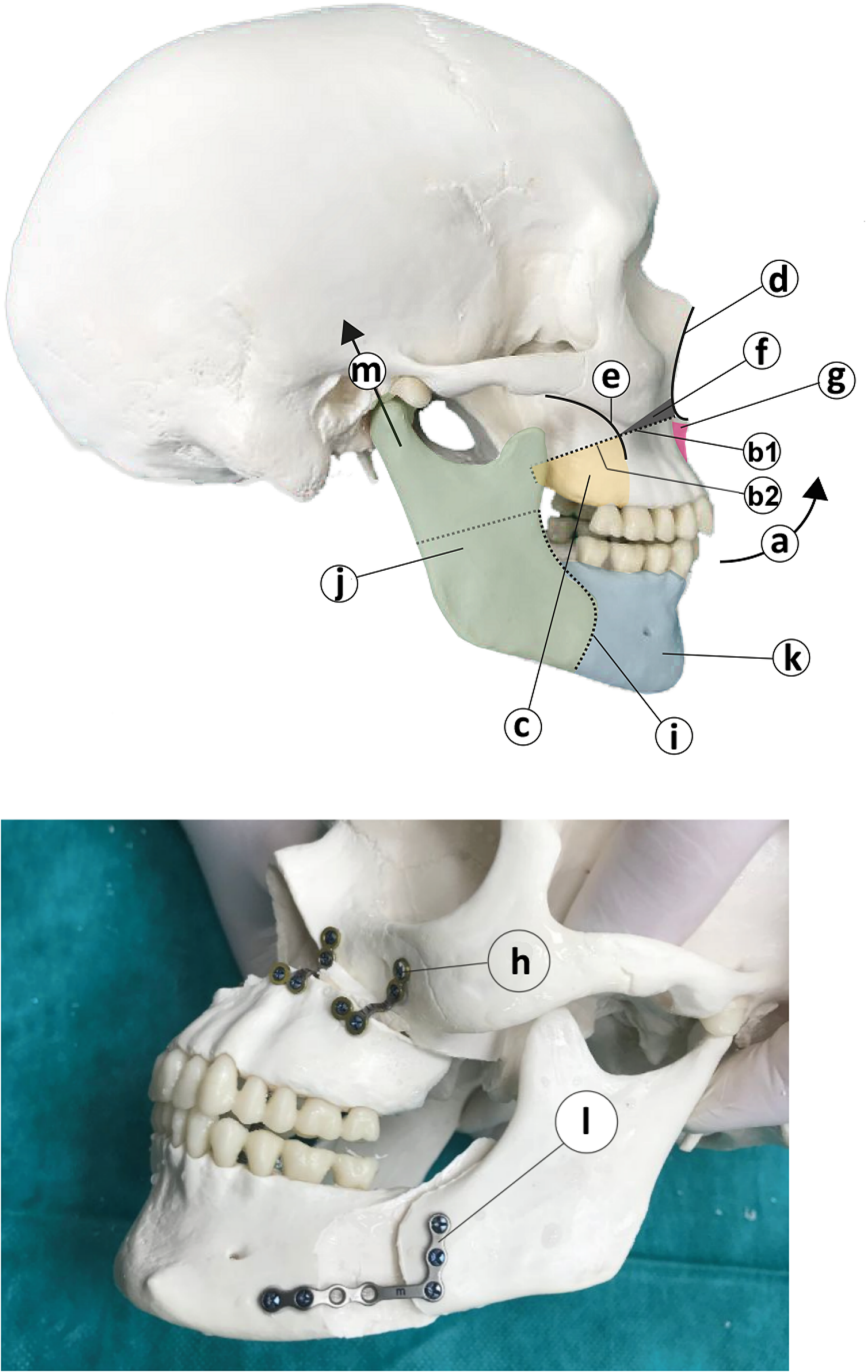
Overview of anatomical structures and landmarks related to the BRA surgery procedure. **a.** Bimaxillary advancement procedure combined with a counter clockwise rotation of the jaws. **b.** A modified Le Fort I osteotomy: resection of a cuneiform fragment of the anterior maxilla part is performed from the alveolar-zygomatic arch to the apertura periformis (**b1**), while the posterior part behind the alveolar-zygomatic-arch is cut through without resecting a bone fragment (**b2**). **c.** The posterior part behind the zygomatic-alveolar arch. **d.** Piriformis aperture. **e.** The zygomatic-alveolar arch **f.** Resected cuneiform fragment of the anterior part of the maxilla with the maximum height at the piriformis aperture descreasing to a minimum at the arcus zygomato-alveolaris. **g.** V-shaped osteotomy of the anterior nasal process. **h.** L-shape mini ostheosynthesis plates. **i.** Obwegeser-Dalpont osteotomy. **j.** Posterior part of the mandible. **k.** Anterior part of the mandible. **l.** Semirigid osteosynthesis miniplate. **m.** The articular process of the anterior part of the mandible positioned in central position with a posterior and cranial alignment.

All patients have been treated using the well-known bimaxillary advancement procedure combined with a counter clockwise rotation of the jaws, see Fig 1(a). In the first step, the mobilization of the maxilla is done with a slightly modified Le Fort I osteotomy (Fig 1(b)). In particular, the anterior part of the maxilla from the piriformis aperture back to the zygomatic-alveolar arch is resected as a cuneiform fragment with the maximum height at the piriformis aperture decreasing to a minimum at the arcus zygomato-alveolaris (Fig 1(b1)). In contrast, the posterior part behind the alveolar-zygomatic-arch is cut through without resecting a triangular bone fragment (Fig 1(b2)). This way the center of maxilla rotation is effectively shifted to its middle point which allows to perform rotation without losing the intermaxillar height. To avoid compression of the nasal septum, a V-shaped osteotomy of the anterior nasal process is performed. An anterior vascularized transposition of at least 5mm with counter clockwise rotation is fixed with 4 L-shape mini ostheosynthesis plates and each plate is fixed with 4 mini screws. As a next step, the mandible is split using the well known Obwegeser-Dalpont osteotomy technique on both sides. The posterior part of the mandible containing the articular process is positioned in central position with a posterior and cranial alignment. The anterior part is fixed to the maxilla using a occlusal splint. Subsequently, the two parts are fixed with a semirigid osteosynthesis miniplate secured with 4-5 miniosteosynthesis screws. The predefined jaw displacements and rotations are used as boundary conditions for subsequent computation of soft tissue deformations. Fig 1 gives an overview of anatomical structures and landmarks related to the BRA surgery procedure.

### Generation of 3D patient models from CBCT and optical surface scanning data

**Fig 2:**
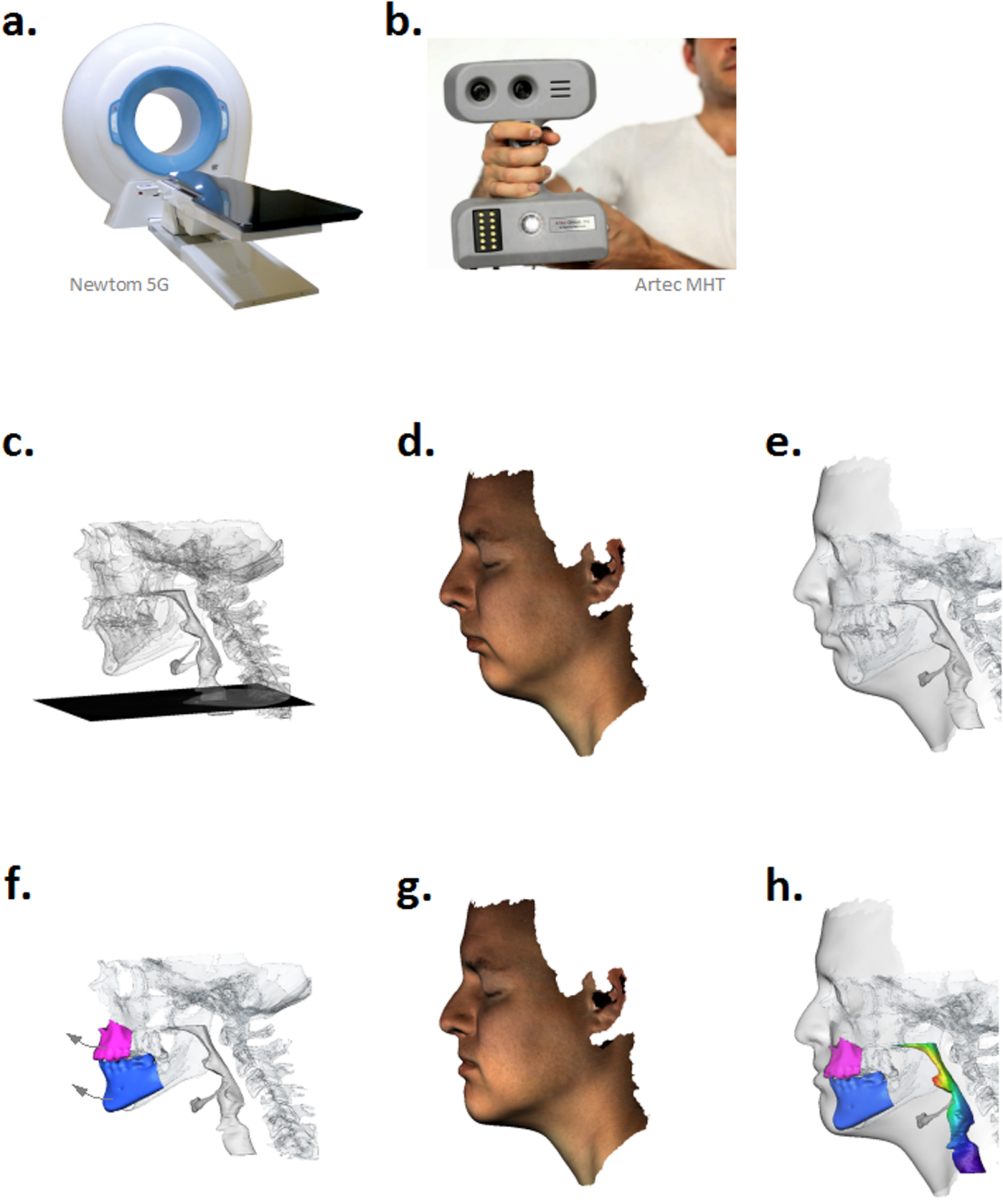
Overview of 3D BRA modeling and simulation pipeline. **a**. CBCT data acquisition is performed using the Newtom 5G scanner (QR S.r.l., Verona, Italy). **b**. Optical surface scanning of patient’s facial contours is performed using the Artec MHT mobile scanner (Artec Inc., Luxembourg). **c**. 3D anatomical model, including soft tissue, bones and airways, is generated from pre-surgical CBCT data. **d**. 3D photo-textured model of patient’s facial contours is generated using Artec Studio v.0.7.4.2 software. **e**. 3D anatomical (**c**) and surface (**d**) models are merged to a single triangulated model. **f**. 3D bone displacement is simulated on the basis of the initial BRA plan. FE simulation is performed to predict post-surgical facial contours (**g**) and extension of airways (**h**) resulting from maxillo-mandibular displacements (**f**).

Cone Beam Computer Tomography (CBCT) data of the patients’ head were acquired in supine position as 512x512x539 (0.3x0.3x0.3mm) DICOM images using Newtom 5G scanner (QR S.r.l., Verona, Italy). DICOM images are semi-automatically segmented and mesh models of facial soft tissues, airways and bony structures are generated using Amira 4.1 software (Mercury Computer Systems Inc., San Diego, USA). Optical surface (OS) scanning of patient’s facial contours was performed in upright position using the Artec MHT mobile scanner followed by generation of a photo-textured surface model in Artec Studio v. 0.7.4.2 software (Artec Inc., Luxembourg). In order to ensure a consistent alignment of volumetric and surface data, CBCT and OS scanning was performed in standard anatomical positions, i.e., the Frankfort line aligned to the vertical (CBCT) or horizontal (OS) axis, respectively. Furthermore, patients were advised to bring upper and lower teeth into occlusion during CBCT and OS scanning in order to normalize the relative mandible location in supine and upright positions. Surface models derived from CBCT and OS data were merged to a single 3D surface model of patient’s head which was subsequently filled with a volumetric (unstructured tetrahedral) grid for computational simulation of soft tissue mechanics using the Finite Element Method (FEM). Fig 2(a)–2(e) presents an overview of the essential steps of 3D data acquisition and processing.

### Computational modeling of hard/soft tissue mechanics

3D anatomical structures are categorized in three basic types of materials: (i) rigid bones (i.e., skull, spine, jaws, hyoid), (ii) deformable soft tissue (i.e., skin, fat, muscles) and (iii) air (i.e., exterior, airways, nasal cavities). Relocated fragments of upper and lower jaw surfaces were segmented for assignment of boundary displacements, see Fig 2(f). To calculate deformation of soft tissue from the predefined displacement of jaws the elastomechanical FEM was used. A detailed description of the FE model used in this work has been reported in [24]. Briefly, the piecewise isotropic, homogeneous, non-linear elastic model based on the generalized Hookean law is used for approximation of constitutive soft tissue properties

$$\begin{array}{c} \sigma \left(\varepsilon \right)=\frac{E}{1+\nu }\;(\varepsilon +\frac{\nu }{1-2\nu }\text{tr}(\varepsilon )I)\;, \end{array}$$ (1)

where ***σ*** is the so-called Cauchy stress tensor, *ε* is the Green-Lagrange strain tensor and (*E*, *ν*) are the Young’s modulus and the Poisson’s ratio—two elastic constants describing material stiffness and compressibility, respectively. Our previous studies have shown that this model is capable to accurately describe the deformation of facial soft tissue in context of cranio-facial surgery planing. Here, we extend this FE framework to prediction of patients’ photo-realistic appearance and pharyngeal airways, see Fig 2(f)–2(h).

### Evaluation of facial and pharyngeal soft tissue prediction

Prior to comparative measurements, pre-/post-surgical and simulated 3D anatomical models are aligned using the Artec Studio rigid registration tool which relies on a set of manually defined landmarks. For quantification of differences between facial and pharyngeal surfaces, the surface distribution of maximum shortest bijective distances between each two surfaces (*A*, *B*), i.e., pre-, post-surgery and simulated facial outlines, is calculated:

$$\begin{array}{c} d_{\text{min}}=\text{max}\;(\text{min}(A\to B),\text{min}(B\to A))\;. \end{array}$$ (2)

**Fig 3:**
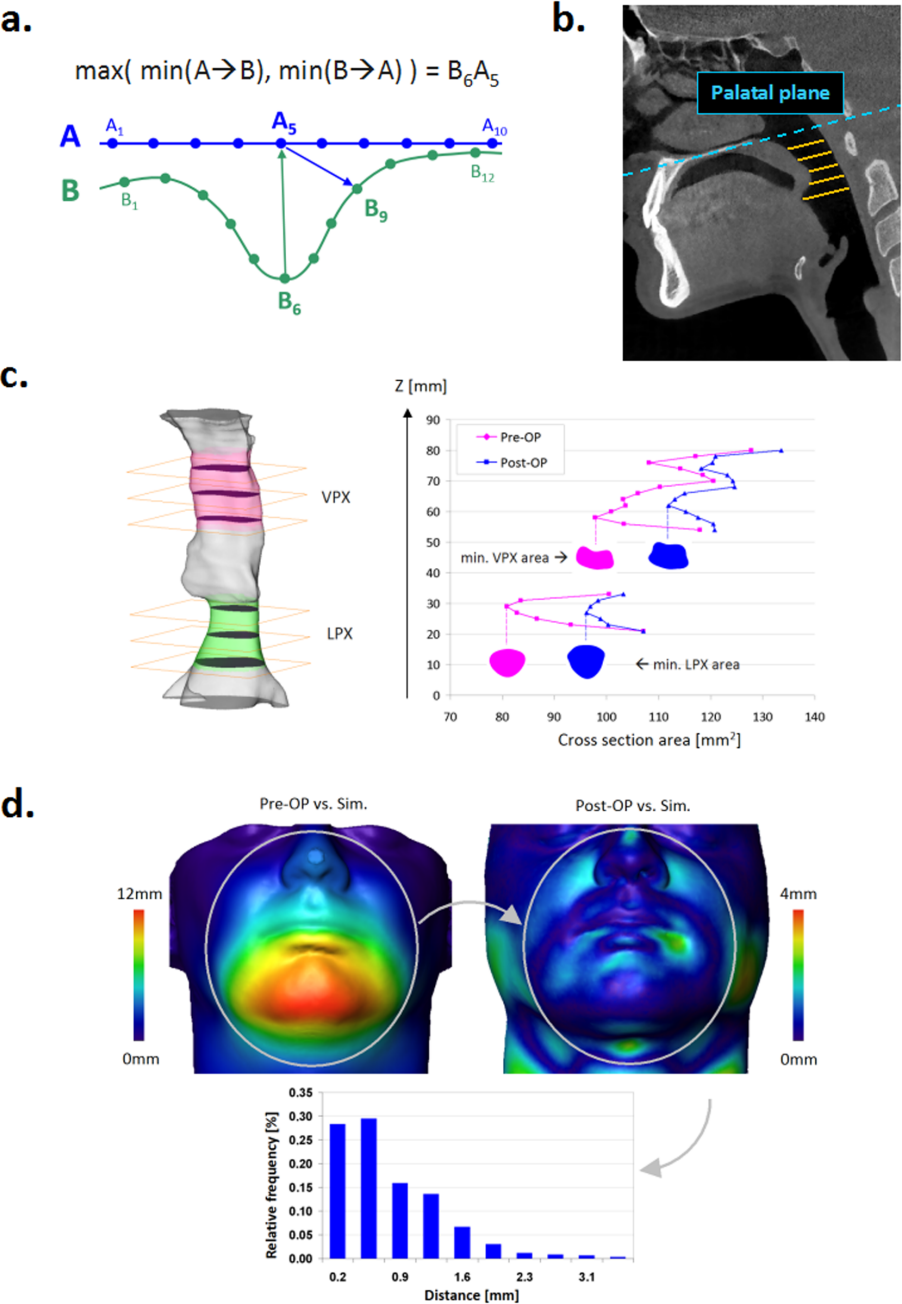
Evaluation of computational soft tissue prediction. **a**. The bijective distance between two surfaces is calculated using the *d*_min_ metrics (Eq 2). The shortest distance between the node *A*_5_ of the surface *A* to and from the surface *B* is given by *A*_5_*B*_9_ and *B*_6_*A*_5_, respectively. Given *B*_6_*A*_5_ > *A*_5_*B*_9_, the *d*_min_ bijective distance between *A* and the surface *B* measured from the node *A*_5_ is *B*_6_*A*_5_. **b**. Morphometric measurements of pharyngeal airways is performed using equidistantly placed cross sections parallel to the palatal plane. **c**. For comparison of post-surgery and simulated airways, the entire set of velo- and laryngopharyngeal cross sections as well as their minimal values are used. **d**. To reduce the effects of geometrical noise, distances between post-surgery and simulated facial surfaces are evaluated using the *d*_min_ metrics in the relevant facial regions, where the magnitude of computationally predicted displacements exceeds the threshold of 0.1mm. (Ellipses indicating the relevant facial regions are shown only for illustration of the principle. The relevant surface patches vary in shape and size depending on the individual distribution of displacements).

The *d*_min_ metrics is introduced to avoid artificially short distances between two convex surfaces when using the unidirectional distance, see Fig 3(a).

To assess changes in geometry of pharyngeal airways, areas and axial dimensions (e.g., anterior-posterior (AP) and lateral (Lat) diameters) of 1mm equidistantly placed cross sections are measured parallel to the palatal plane, see Fig 3(b). For analysis of changes between pre-/post-surgery and simulated airways, comparison of velo- and laryngopharyngeal cross sections using the t-test is performed. Furthermore, differences in areas and AP/Lat dimensions of the narrowest cross section are assessed Fig 3(c).

For analysis of differences between facial surfaces, the distribution of *d*_min_ values is assessed for surface nodes, where the magnitude of computationally predicted displacements exceeds the threshold of 0.1mm. This way analysis of surface distances is naturally restricted to the relevant facial regions such as largely deformed chin, chick, lips, nose areas. Irrelevant regions with the vanishing magnitude of simulated displacement are excluded from analysis, see Fig 3(d). For each patient, the surface distribution of *d*_min_ is analyzed in terms of mean and stdev values. The individual *d*_min_ averages are subsequently used for calculation of cohort *d*_min_ mean and stdev values.

## Results

Computational simulation of soft tissue deformation upon BRA treatment is performed for 10 OSA patients using pre-surgery 3D image data and the elastomechanical FE simulation as described above. An example of simulated impact of BRA on facial and pharyngeal soft tissues in a 37 y.o. male patient (case study #1 in Table 1) is shown in S1 and S2 Movies.

**Fig 4:**
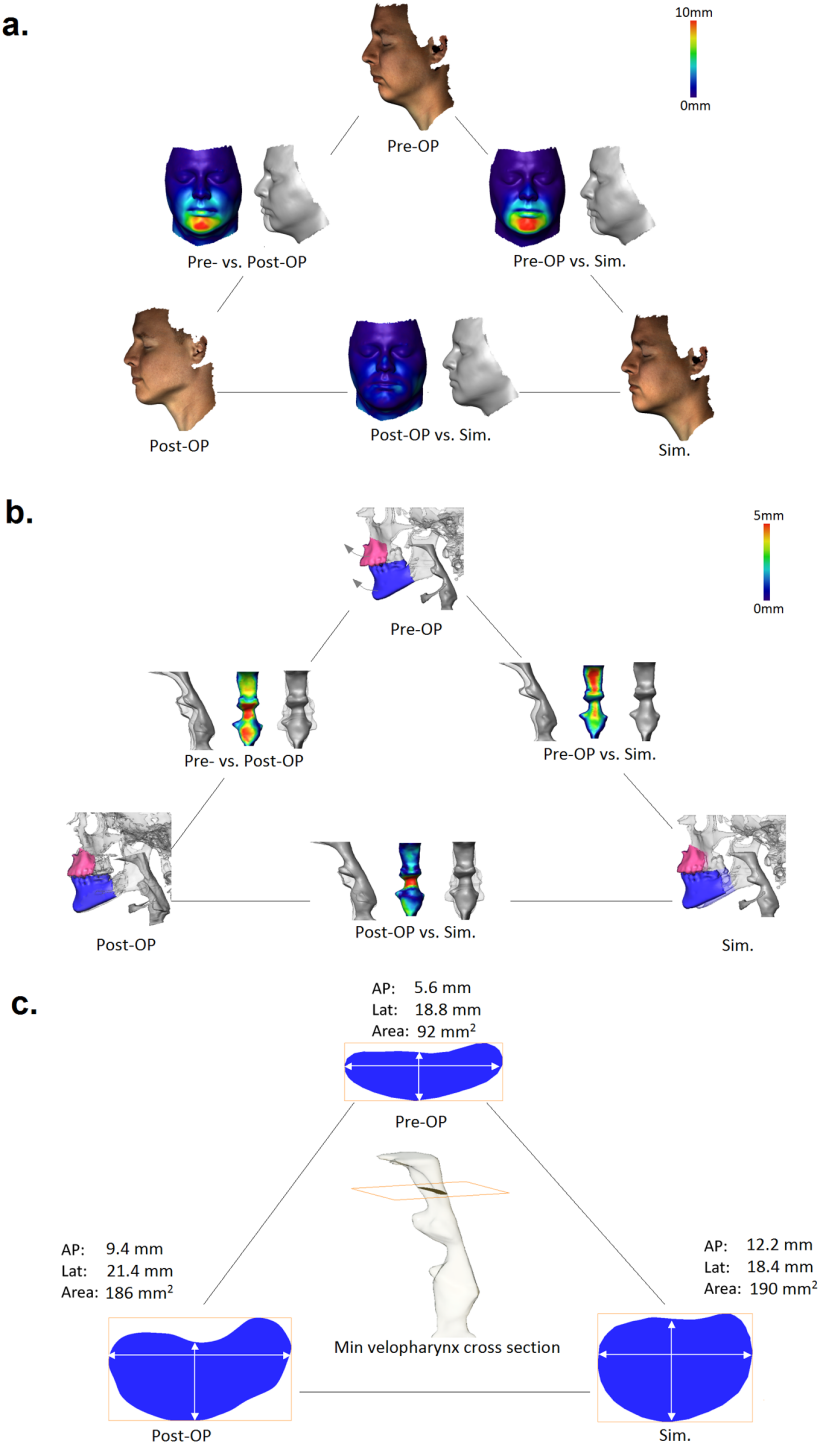
Case study #1: comparison of (**a**) pre-/post-surgical vs simulated facial surfaces, (**b**) pharyngeal airways, (**c**) the narrowest velopharyngeal cross section area and linear dimensions (AP,Lat) for a 37 y.o. male OSA patient underwent BRA treatment consisting of maxilla protrusion (5mm) / impaction (-3mm) and mandibula protrusion (12mm).

**Fig 5:**
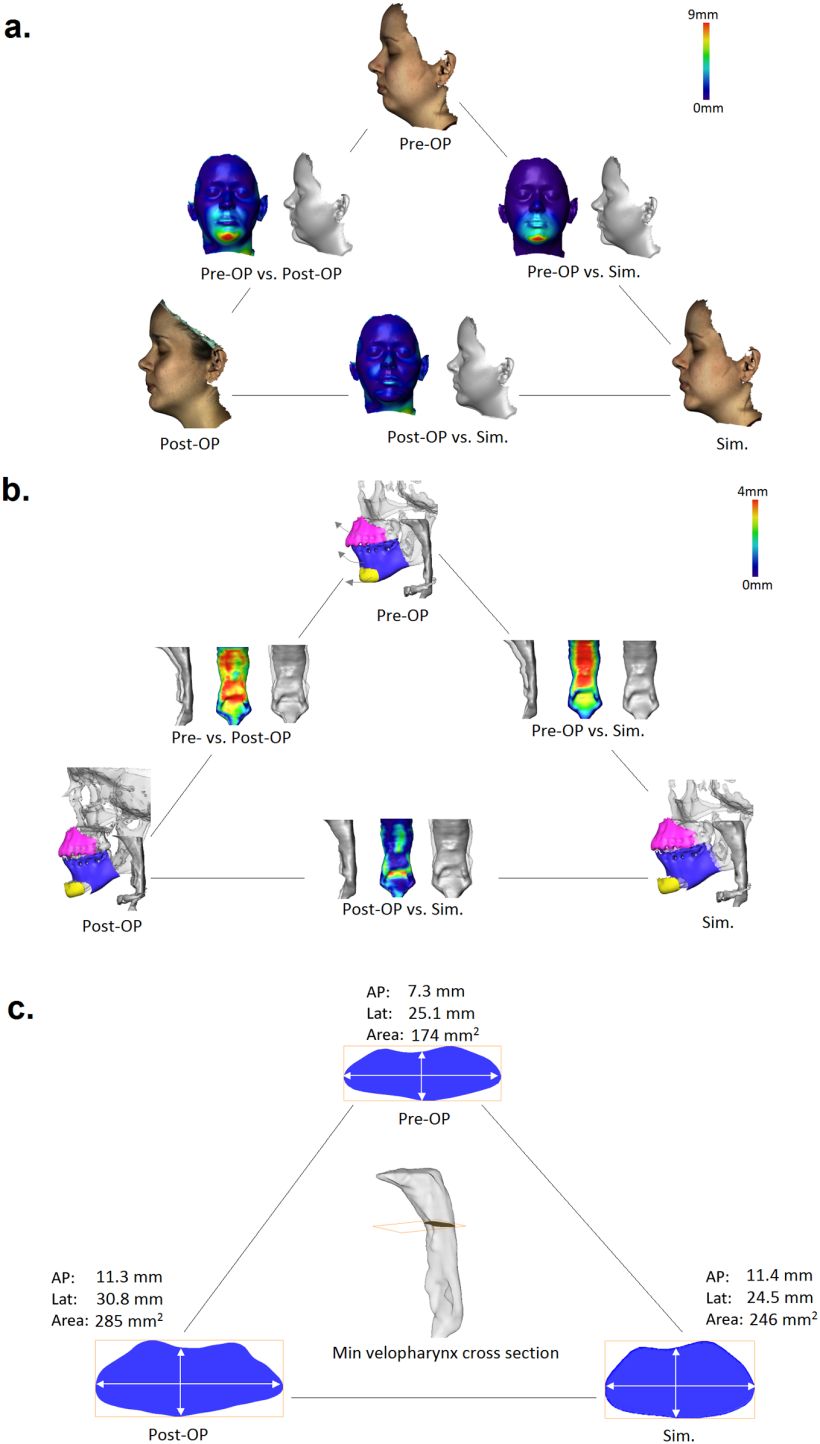
Case study #2: comparison of (**a**) pre-/post-surgical vs simulated facial surfaces, (**b**) pharyngeal airways, (**c**) narrowest velopharyngeal cross section area and linear dimensions (AP,Lat) for a 25 y.o. female OSA patient underwent BRA treatment consisting of maxilla protrusion (3mm) / impaction (-3mm) and mandibula protrusion (9mm) with subsequent chin advancement.

To validate accuracy of facial soft tissue predictions, the maximum shortest bijective distance (*d*_min_) between each two facial surfaces is calculated using Eq 2 as described in Fig 3(a) and 3(d). Figs 4 and 5(a) show examples of crosswise distance maps between pre-, post-surgery vs. simulated facial surfaces including color-mapping of *d*_min_ values. Mean and stdev values of individual *d*_min_ distributions are summarized in Table 2. Our experimental data show that deviation of computationally predicted facial surfaces from post-surgery results amounts in group average to 0.8mm (SD = 0.7mm). The largest deviation of model predictions from post-surgery data is found in interface regions between the nose, lips and bones, where special boundary conditions such as tissue-bone sliding occur.

**Table 2:** Comparison of post-surgery and simulated soft tissue outcome including average *d*_min_ distances (Eq 2) between facial surfaces and dimensions of the narrowest cross section of velo- and laryngopharyngeal regions.

	Face post/sim		Min. VPX cross section						Min. LPX cross section					
	d_min_ [mm]		A [cm^2^]		AP [cm]		Lat [cm]		A [cm^2^]		AP [cm]		Lat [cm]	
N	mean	std	post	sim	post	sim	post	sim	post	sim	post	sim	post	sim
1	0.4	0.3	1.9	1.9	1.0	1.2	2.1	1.9	4.9	4.3	2.5	2.4	3.2	2.4
2	0.3	0.3	2.9	2.5	1.1	1.1	3.1	2.5	4.3	2.6	1.9	1.6	3.1	2.6
3	1.8	1.6	1.3	1.3	0.8	1.1	2.4	1.4	4.5	4.0	1.7	1.9	3.7	3.3
4	0.5	0.6	1.6	0.9	0.9	0.9	1.9	1.2	4.2	3.7	2.3	2.5	3.2	3.1
5	1.8	1.4	5.2	4.1	1.8	1.6	3.5	2.8	3.1	2.4	1.8	1.9	2.8	2.0
6	0.3	0.4	1.5	1.4	0.9	0.9	2.0	1.5	2.9	2.2	1.6	1.5	2.7	2.2
7	2.1	1.7	2.9	2.1	1.2	1.2	2.8	2.0	4.1	3.5	2.4	2.2	3.2	1.9
8	0.4	0.5	1.3	0.8	0.9	0.8	1.7	1.1	1.9	2.2	1.4	1.2	1.7	1.5
9	0.3	0.4	3.6	2.9	1.4	1.2	3.3	2.4	5.5	5.0	1.8	1.7	3.9	3.5
10	0.4	0.3	1.7	1.1	0.8	0.7	2.4	1.6	0.9	0.7	0.9	0.9	1.3	1.0
mean	0.83	-	2.4	1.9	1.1	1.1	2.5	1.8	3.9	3.3	1.9	1.9	3.1	2.5
std	0.75	-	1.3	1.0	0.3	0.3	0.6	0.6	1.4	1.3	0.5	0.5	0.8	0.8
min	0.30	-	1.3	0.8	0.8	0.7	1.7	1.1	0.9	0.7	0.9	0.9	1.3	1.0
max	2.10	-	5.2	4.1	1.8	1.6	3.5	2.8	5.5	5.0	2.5	2.5	3.9	3.5

For comparison of pre-, post-surgery and simulated airways, *d*_min_ distances between registered pharyngeal surfaces are computed. Figs 4 and 5(b) illustrate 3D superpositions and color-mapping of distances between pre-, post-surgery and simulated pharyngeal airways. While pre/post and pre/sim distance maps qualitatively show expected differences in velopharynx, large post/sim deviations are observed in oropharyngeal region. We trace these deviations back to higher geometrical variability of the oropharyngeal region due to occasional swallowing artifacts and different tongue positions in pre- and post-surgery 3D scans. To quantify the overall differences between pre- and post-surgery pharyngeal airways upon BRA and to evaluate agreement between post-surgical and simulated outcomes, areas and AP/Lat diameters of equidistantly placed VPX and LPX cross sections in the palatal plane are measured. The results of statistical testing of dissimilarity between the entire set of cross section areas and AP/Lat diameters using the two-paired t-test are summarized in Table 3. As one can see, computational simulation provides a quantitatively good estimate for post-surgery changes in VPX cross section areas and AP diameters, i.e., low- or non-significant sim/post difference, but fails to accurately describe Lat stretch of post-surgical VPX cross sections. For the LPX region, lower significance of pre/post differences as well as lower accuracy of computational predictions is measured.

**Table 3:** Significance of differences between cross section areas and AP/Lat diameters of pre-/post-surgery and simulated velo- and laryngopharyngeal airway regions.

	Area			AP diameter			Lat diameter		
VPX,p-val	***	***	*	***	***	n.s.	***	n.s.	**
LPX,p-val	**	***	**	**	***	**	*	n.s.	*

***: p<0.001,

**: p<0.01,

*: p<0.05,

n.s.: p>0.05

**Fig 6:**
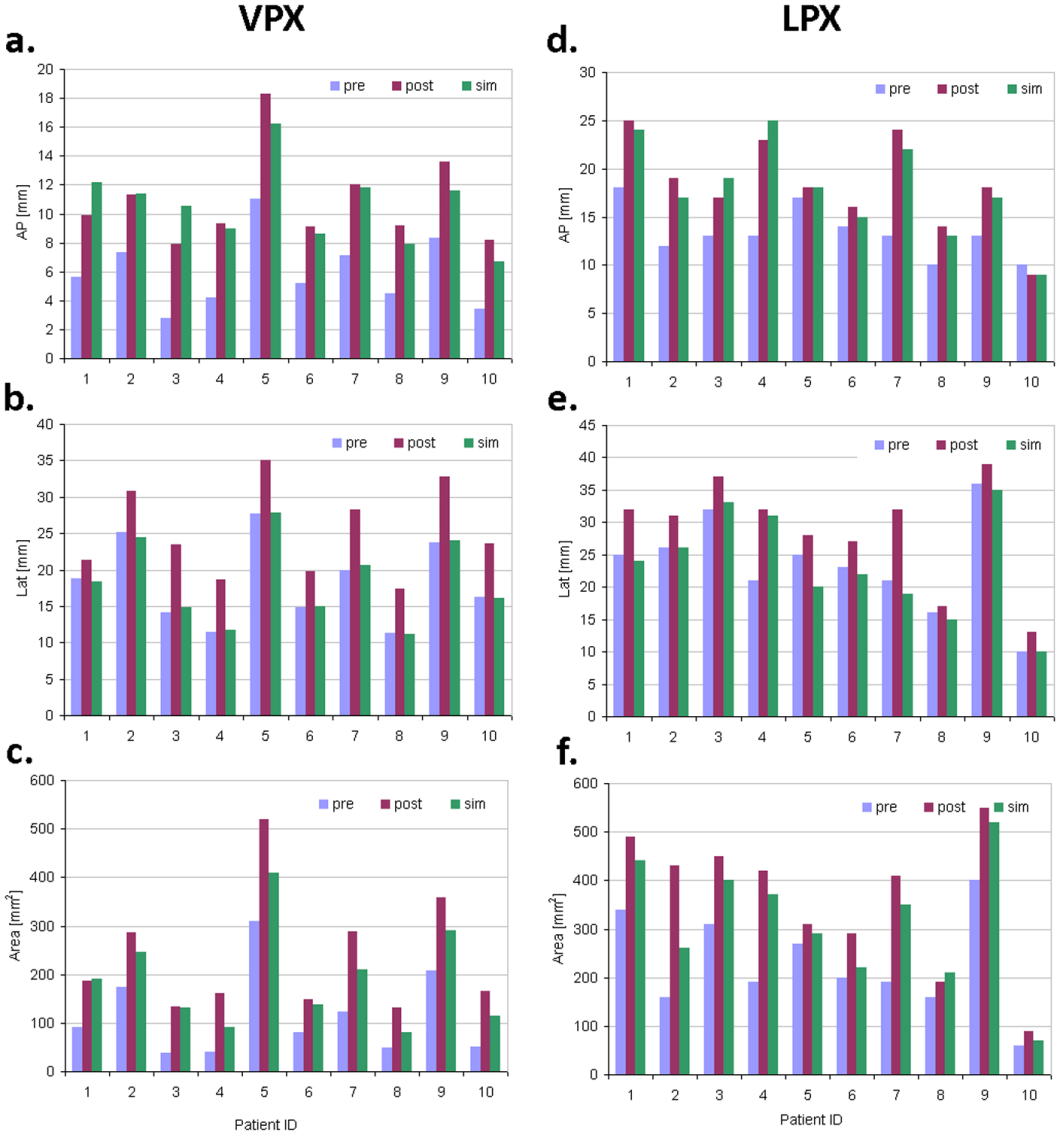
Comparison of antero-posterior (**a,d**), lateral (**b,e**) diameters and cross section areas (**c,f**) of the narrowest VPX and LPX cross sections between pre-, post-surgery data and computational simulation.

Based on repeatedly reported physiological relevance of pharyngeal constrictions for emergence of OSA symptoms [16, 25–30], the narrowest VPX and LPX cross sections are identified. Figs 4 and 5(c) show examples of post-surgery and simulated extension of the pre-surgery narrowest velopharyngeal cross sections. Remarkably, our FE model provides a comparatively accurate estimate for AP extension of constricted VPX pharyngeal regions, which deviation from post-surgery result amounts in cohort average to 12% (SD = 11%), see Fig 6(a). In contrast, the Lat diameter of the narrowest VPX cross sections exhibits in average 30% (SD = 10%) larger stretch in post-surgery data than estimated by the FE simulation, see Fig 6(b). Accordingly, the cross section area predicted by FE model is typically smaller than the post-surgical result, Fig 6(c). Similar differences between post-surgical and simulated cross sections are found for the LPX region: computational model provides a more accurate prediction for the AP (Fig 6(d)) than for the Lat diameter (Fig 6(e)) and the cross section area (Fig 6(f)).

Post-surgical polysomnogram measurements reveal a consistent reduction of AHI to more than 50% of its pre-surgical value for almost all participants, while the absolute values of post-surgery AHI in this cohort exhibit a large variability in the range between 0.5–36.6 with mean±stdev = 11.3 ± 11.6.

## Discussion

Experimental results of this study confirm previous observations [24] of a sufficiently accurate prediction of post-surgical facial appearance using an isotropic, homogeneous constitutive model of soft tissue mechanics. This can be explained by a particular organization of anisotropic facial soft tissue forming a relatively thin, quasi-2D layer which has a surface tangent that is nearly perpendicular to the direction of skin displacement triggered by bimaxillary advancement with counterclock rotation.

Structural organization of pharyngeal soft tissue does not exhibit such an exceptional symmetry which leads to more pronounced anisotropic effects and, consequently, larger deviation of computational predictions from post-surgery deformation of pharyngeal airways. Large lateral elongation of the post-surgical velopharynx has been previously reported in the literature [11, 14]. However, its mechanism is not yet well understood. It is reasonable to assume that the lateral stretch of pharyngeal and, in particular, velopharyngeal airways is caused by pharyngeal muscles that establish a remote mechanical link between displaced jaws and airway walls. The transmission of forces along muscle fibers is associated with reduced dissipation of mechanical energy in comparison to an isotropic, homogeneous medium assumed in our material model. To account for anisotropic effects of pharyngeal muscles, consideration of anisotropic properties of muscle fibers is required. Since generation of individual anisotropic models is not feasible, utilization of anisotropic templates has been previously suggested [31]. Anisotropic templates of highly complex pharyngeal musculature are, however, widely missing. Alternatively, effects of pharyngeal muscles can be simulated by introducing corrective (penalty) forces that will account for differences between post-surgical airways displacements and displacements obtained from computational simulation using the simplified anisotropic model.

## Conclusion

Complexity of the head anatomy and mechanical tissue behavior makes quantitative planning of bimaxiallary advancement for treatment of OSA a challenging task. Reliable computational models of facial and pharyngeal soft tissue are highly demanding for the prediction of functional and esthetic BRA outcome. Comparison of our simulation results with post-surgical data indicates that our soft tissue model is capable to estimate facial tissue displacements and antero-posterior extension of pharyngeal space upon BRA. Thereby, largest deviations of model predictions from post-surgical data are observed in the lips, nose and velopharyngeal regions. We trace these deviations back to special boundary conditions (such as tissue-bone sliding) and anisotropic properties of pharyngeal muscles that are not considered by our piecewise isotropic homogeneous soft tissue model. Consideration of anisotropic effects of pharyngeal muscles can also be studied on post-surgery relocation of the hyoid bones which is known to be a natural mediator and landmark of pharyngeal muscles action [32].

Our computational framework provides low bound estimation for post-surgical extension of pharyngeal airways. A physically consistent simulation of resulting effects on pharyngeal airflow is principally feasible, however, it requires additional assumptions of material parameters of the patient’ soft tissue such as elasticity of airways walls and anisotropy of pharyngeal muscles. Further experimental and computational studies are required to determine whether obvious features of airway geometry, such as dimensions of the narrowest velopharyngeal cross section, or more subtle physical indicators of local airflow instability can provide robust criteria for optimal planning of OSA treatment using BRA.

## Supporting information

S1 Movie Example of simulated BRA impact on facial soft tissues and esthetic appearance of a 37 y.o. male OSA patient (case study #1 in Fig 6(a)). (AVI)

S2 Movie Example of simulated BRA impact on pharyngeal airways in a 37 y.o. male OSA patient (case study #1 in Fig 6(a)). (AVI)
